# An Update on the Changing Indications for Androgen Deprivation Therapy for Prostate Cancer

**DOI:** 10.1155/2011/419174

**Published:** 2011-02-07

**Authors:** Kristene Myklak, Shandra Wilson

**Affiliations:** Division of Urology, Department of Surgery, University of Colorado Denver, Aurora, CO 80045, USA

## Abstract

Quality of life has become increasingly more important for men diagnosed with prostate cancer. In light of this and the recognized risks of androgen deprivation therapy (ADT), the guidelines and use of ADT have changed significantly over the last few years. This paper reviews the current recommendations and the future perspectives regarding ADT. The benefits of ADT are evident neoadjuvantly and adjuvantly in patients treated with external beam radiation therapy for intermediate- and high-risk disease, in patients who have undergone prostatectomy with lymph node involvement, in high-risk patients after definitive therapy, and in patients who have developed progression or metastasis. Finally, this paper reviews the risks and benefits of each of these scenarios and the risks of androgen deprivation in general, and it delineates the areas where ADT was previously recommended, but where evidence is lacking for its additional benefit.

## 1. Introduction

Prostate cancer is the most frequently diagnosed cancer in males in the United States and has long been associated with hormone dependence [1]. The use of androgen deprivation therapy (ADT) for men with advanced prostate cancer continues to be the recommended therapy. Androgen deprivation is defined as a lowering of serum testosterone through the administration of a luteinizing hormone releasing hormone (LHRH) agonist. However, it has become increasingly apparent that ADT is not without its own risks. ADT-associated risks continue to become more fully elucidated through multiple recently published retrospective and prospective studies. These risks are no longer solely defined by life span and cancer progression but also by how ADT affects quality of life based on a patient's physical, financial, and emotional well being. There is also evidence that a “middle of the road”, intermittent androgen deprivation therapy (IADT) may soon be appropriate care for some individuals with prostate cancer. This paper examines IADT and the clinical studies that are being done that suggest it as a possible alternative in the future. This paper also reviews the findings of investigations into the risks and benefits of ADT. It will delineate areas where ADT use has been deemed inappropriate/ineffective and summarizes the current clinical situations where ADT use remains recommended.

## 2. Androgen Deprivation and Associated Adverse Events

### 2.1. Cardiovascular Disease

ADT utilizes the fact that malignant prostate cells require androgen stimulation for growth and division. ADT attempts to deny malignant cells a growth stimulus, potentially resulting in a slowing of cancer growth and progression. However, androgen receptors are not located only within the prostrate. Androgen receptors are known to be expressed in endothelial cells and have been shown to regulate a number of endothelial responses [2]. ADT has been shown to lead to an increase in cardiovascular disease that correlates with an increase in myocardial infarction and even sudden cardiac death in some studies. Keating et al. found that men with prostate cancer using a GnRH agonist were associated with an increased risk of coronary heart disease (adjusted hazard ratio (HR), 1.16; 95% confidence interval (CI), 1.10 to 1.21), myocardial infarction (adjusted HR, 1.11; 95% CI, 1.01 to 1.21), and sudden cardiac death or life-threatening ventricular arrhythmia (adjusted HR, 1.16; 95% CI, 1.05 to 1.27). This increased risk of coronary heart disease was evident in as few as one to four months [3]. It is now well understood that the decreased lean body mass and increased body fat composition seen grossly with the administration of ADT [4] are also correlated with negative changes in the serum lipid profile, an increased risk of insulin resistance and an increased risk of coronary artery disease. A twelve-month study of 40 men with prostate cancer demonstrated that ADT increased serum total cholesterol by 9 percent, low-density lipoprotein cholesterol by 7 percent and triglycerides by 27 percent [5]. This risk of the development of diabetes and coronary artery disease has been confirmed in other studies and appears to be even greater in men over 65 years of age [6]. In a 3-month prospective study of nondiabetic men, ADT significantly increased fasting plasma insulin by 26 percent and decreased insulin sensitivity by 13 percent [7]. Description of this “metabolic syndrome” and the concern about its impact on survival on patients treated with ADT led to the reanalysis of the RTOG 92-02 trial. In this trial, 1554 men with locally advanced prostate cancer were treated with neoadjuvant goserelin for 4 months prior to radiation therapy and either no additional therapy or for 24 additional months. Although a striking increase in death from coronary artery disease was not seen in the men who underwent prolonged treatment with ADT, the authors did note that “although there was a significant advantage for all prostate cancer-specific endpoints (with prolonged therapy), the longer-term arm of ADT in RTOG 92-02 was associated with greater noncancer mortality than [the] short-term [arm].” They go on to state that “compared to the general population, men with prostate cancer (are known to) have higher rates of noncancer death and GnRH agonists may contribute to this through multiple mechanisms” [8]. An interim analysis of a recent prospective randomized trial showed a reversal of the negative effects on low density lipoprotein (LDL), very low density lipoprotein (VLDL), and high density lipoprotein (HDL) with the administration of toremifene [9]; a similar effect would likely be seen with 3-hydroxy-3-methylglutarylcoenzyme A reductase inhibitors or “statins.” It is recommended at this time to screen carefully and treat all patients on ADT for hyperlipidemia, diabetes, and coronary artery disease [10]. 

### 2.2. Skeletal Disease

Men undergoing ADT have an increased risk of developing osteoporosis due to androgen deprivation. Osteoporosis onset is early and associated with a decrease in bone mineral density leading to an increase risk of bone fracture [11]. Several studies and research have been underway to investigate the best treatment at both preventing and treating ADT-associated fractures. It is recommended that all men over 50, and particularly those treated with ADT be supplemented with 800 to 1000 IU of vitamin D, 1200 mg of calcium daily, frequent weight-bearing exercise, and to be screened for osteoporosis regularly [12]. Those at higher risk for fracture should be treated with bisphosphonates. A prospective randomized study of alendronate (70 mg weekly by mouth versus placebo) started at the initiation of ADT showed improvement of spine and hip densities, and this finding was less significant it was initiated after just one year of ADT [13]. Recent studies have supported findings that 60 mg denosumab, a monoclonal antibody to RANKL, twice yearly helps prevent and treat ADT-induced bone loss and fractures [14]. Quarterly or annual 4 mg zoledronic acid infusions for men with normal creatinine show similar or even greater benefits, although the rare risk of osteonecrosis can be devastating [13, 15, 16]. It is the recommendation of the National Comprehensive Cancer Network that zoledronic acid be administered to men with bony progressive metastatic prostate cancer on ADT to help prevent skeletal related events.

### 2.3. Quality of Life: Cognition and Physical Function

Cognition and physical function are important factors for how a patient defines quality of life, and there is increasing evidence that ADT may have adverse effects on these functions as well. A retrospective trial showed that up to 27% of patients on ADT suffered a diagnosable psychiatric illness during their treatment, and that, in patients on ADT tested over time, many lost cognition in one, if not two, measurable areas [17, 18]. However, ADT directly resulting in permanent cognitive dysfunction remains controversial. A recent prospective controlled study found no consistent evidence that after one year of ADT use there is declining cognitive function [19]. Additional studies suggest that while there may be some cognitive dysfunction with ADT use, cognition may return to baseline with cessation [20]. A recent prospective controlled study of men on androgen deprivation more than 6 months found a decline of lower body physical function with a statistically slower walk and chair-rise times with treatment [21]. A retrospective study from the Mayo Clinic in Scottsdale found that the average hemoglobin drop on patients treated with LHRH antagonists is 1.6 g/dL, which may contribute to the physical decline seen in these patients [22]. Additional studies have documented decreases in hemoglobin concentration, Smith et al. noted that eighteen of 22 (82%) men with baseline hemoglobin concentrations 13.5 g/dl or greater developed anemia during ADT [5]. As previous studies have found a profound and prolonged suppression of testosterone long after the cessation of LHRH analogues (53% of men who had been on ADT for 4 or more years remain castrate for up to 2.5 years) [23], there is concern that the adverse risks of coronary artery disease, diabetes, osteoporosis, cognitive/physical changes, and anemia could persist beyond active therapy resulting in long-term impacts on quality of life. Identifying the risks of ADT is important because individuals with prostate cancer should be made aware of the effects of treatment on their quality of life [24]. These factors should be assessed during the decision-making process about desired treatment.

## 3. Intermittent ADT

Although it would seem that the financial costs of ADT are minimal, a recent study showed, if used for a significant amount of time, the cost of ADT would quickly be greater than that of radical prostatectomy or external beam therapy [25]. At a time when the cost to the government to cover medical costs for its citizens is rapidly approaching one-half of the United States budget, this is not a trivial issue. IADT would seem to be a “middle of the road” option offering the ability to deliver the proven efficacy of constant ADT while lowering the adverse events and cost. A recent European study found no significant difference in survival between intermittent ADT compared to continuous ADT. The study found that the greater number of cancer deaths in the intermittent arm (106 verses 84) were balanced by the greater number of cardiovascular deaths in the continuous arm (52 verses 41) [26]. A large ongoing phase III trial by Southwest Oncology Group (SWOG) 9346 involves 1500 patients with stage D2 prostate cancer. Subjects were pretreated to a PSA of = 5 ng/mL and underwent a 7-month induction period with goserelin and bicalutamide. Subjects with a stable or declining PSA level of = 4 ng/mL were randomized to continuous ADT or IADT. Patients in the IADT group remained off treatment until PSA began to rise above their baseline levels, >20 ng/mL, or above a point determined by the investigator's discretion. These cycles continued until clinical or PSA progressing appeared [27]. Results are still pending but could demonstrate the long-term utility of IADT. An article update on IADT stated that, “Although evidence suggests that IADT performs at least as well as continuous ADT in terms of survival and perhaps better in terms of side effects IADT still remains experimental and unproven regarding long term implications of disease progression and survival impact.” [28] Studies to date demonstrate that IADT appears to be a suitable alternative to constant ADT with associated decreased costs and improved patient quality of life. A prospective phase II study found that patients receiving IADT reported benefits in the off-treatment interval with a reported increase in quality of life [29]. Further randomized studies will also be required to assess the long-term cardiovascular, skeletal, and cognitive effects of IADT compared to constant ADT. 

Undoubtedly, the adverse risks associated with ADT are justified in a specific subset of prostate cancer patients. However, with the increased awareness about ADT-associated risks coupled with ADT's questionable efficacy, there has been a shift in the recommendation of ADT in several clinical scenarios outlined below.

### 3.1. ADT Is Likely Unwarranted with Low-Risk Localized Disease

#### 3.1.1. Preprostatectomy or Postprostatectomy

Recommendations for neoadjuvant androgen deprivation prior to or after prostatectomy have waned in men with localized disease. Many studies have been completed evaluating ADT efficacy in this cohort and have demonstrated a possible decrease in positive surgical margins; however, there has been no documented improvement in overall survival, making it difficult to justify the risks of ADT [30]. One of the largest prospective randomized prostate cancer trials, the Early Prostate Cancer (EPC) trial, evaluated 150 mg of bicalutamide daily in addition to standard therapy for men with low- and high-risk disease. Analysis of the overall trial did not show any advantage for its use in low-risk patients treated with surgery, radiation, or observation. Although, in general, antiandrogen monotherapy is thought to be inferior and less well tolerated than traditional androgen deprivation [31], the study did suggest some benefit in higher-risk patients with locally advanced or micrometastatic disease in progression-free survival, but no benefit in overall survival was observed [32]. 

#### 3.1.2. Prior to External Beam Radiation/Brachytherapy

Although earlier studies might have suggested some benefit for neoadjuvant ADT prior to external beam radiation for patients with localized disease, with higher dosing [33], conformal techniques that appear to make doses of 70 to 79 Gy possible with minimal toxicity [34], and the wider availability of brachytherapy, it appears that, in general, neoadjuvant androgen deprivation is no longer recommended prior to radiation therapy for patients with low-risk prostate cancer [35, 36]. 

#### 3.1.3. No Primary Treatment

Results of a large retrospective trial recently published in JAMA evaluated 19,271 with localized prostate cancer found no increase in 10-year overall survival in men treated with androgen deprivation compared to conservative management; notably, there was a lower 10-year prostate-cancer-specific survival in men treated with primary androgen deprivation [37]. Similarly, when Dr. Studer reported on the results of the EORTC trial wherein 939 men with prostate cancer not suitable for local curative treatment were evaluated after their randomization to immediate versus deferred ADT, he concluded that “patients with a baseline PSA > 50 ng/mL and/or a PSADT (prostate-specific antigen doubling time) <12 months were at increased risk to die from prostate cancer and might have benefited from immediate ADT whereas patients with a baseline PSA < 50 ng/mL and a slow PSADT (>12 months) were likely to die of causes unrelated to prostate cancer, and thus could be spared the burden of immediate ADT” [38]. The group of men with low-risk tumors who do not get treated with ADT may benefit from active surveillance where similar rates of efficacy are achieved with decreased morbidity compared to definitive therapy or androgen deprivation [39, 40]. Large trials have suggested that there is little benefit in screening for prostate cancer (or at least a large number needed to treat to see benefit), particularly for men over 70. This same cohort that previously would have been treated with ADT due to inability to tolerate definitive therapy may now be likely to avoid screening, diagnosis, and overtreatment with androgen deprivation as well [41, 42]. 

### 3.2. ADT Is Warranted with Advanced Disease

#### 3.2.1. Advanced Disease; PSA > 50, PSADT < 12 Months

Clearly the risks associated with ADT are worth the possible benefit in men with high-stage, high-grade tumors, with rapid recurrence after therapy, doubling times under 12 months and/or total PSA values greater than 50 ng/mL [38]. These men should also have consultation with medical oncology early and should be considered strongly for clinical trials.

#### 3.2.2. Advanced Disease Prior to External Beam Radiation

The administration of neoadjuvant ADT remains an independent predictor of long-term control in patients with intermediate and high-risk cancer treated with external beam radiation (EBXRT) and should be given prior to therapy [43, 44]. A randomized trial of 802 Australian men found that 3 months of neoadjuvant ADT showed an inferior prostate-cancer-specific mortality to men treated with 6 months with locally advanced prostate cancer, so a treatment with 4 to 6 months of neoadjuvant ADT is recommended prior to external beam radiation [45]. The initial finding in the randomized controlled RTOG/EORTC trials showing that an additional 24 to 36 months of ADT after EBXRT improves survival in men with high-risk disease and is recommended as well [46, 47]. In these trials, androgen deprivation has been achieved using an LHRH agonist as well as an antiandrogen. Similar efficacy may be able to be achieved with LHRH monotherapy, but formal comparative studies have not been performed. Interestingly, when a group in Spain looked at predictors for hematuria in patients who had undergone conformal prostate radiation for cancer, the administration of adjuvant androgen deprivation was protective (a factor of 5) for the development of hematuria (whereas transurethral resection [TURP] increased the risk three times) [48]. 

#### 3.2.3. Lymph Node Involvement at Prostatectomy

There is support for immediate ADT in men found to have lymph node involvement at prostatectomy. Only one randomized, controlled study has been performed to investigate this situation to date. Although this trial is small and may have exaggerated survival advantages by delaying the initiation of ADT in the initially untreated group, the study showed a significant improvement in overall, disease-specific, and progression-free survival advantage for patients who undergo early ADT in this setting [49]. 

#### 3.2.4. Local Obstructive Symptoms/Metastatic Disease

Data from the MRC trial published in 1997 showing that ADT helps decrease symptoms from patients with advanced disease is still to be considered today. This prospective randomized trial of 938 men with locally advanced or asymptomatic metastatic prostate cancer showed that rates of pathological fracture, spinal cord compression, ureteric obstruction, the development of extraskeletal metastases (and pain from these metastases) as well as death from prostate cancer are statistically more common in men who were not treated with early androgen deprivation [50]. It is important to note that since the introduction of PSA screening there has been a significant stage migration downward, so patients with this level of disease burden may be less common.

## 4. Adjuvantly in Cryotherapy

In men wishing to undergo primary cryotherapy for prostate cancer, the largest series in the literature reports that ADT used adjuvantly can be successful and may be beneficial with this treatment modality [51]. Recent studies have also suggested that cryotherapy is becoming a safe and feasible treatment for prostate cancer. However, as previously discussed, quality of life after a patient undergoes cryotherapy is important and further multi-institutional studies will be necessary to adequately assess the most appropriate therapy for the patient [52]. Additional studies comparing cryotherapy alone to cryotherapy with ADT will also need to be completed as cryotherapy treatment for prostate cancer advances.

### 4.1. Further Risk Stratification Is Warranted

#### 4.1.1. Biochemical Recurrence after Therapy

Data following the natural course of men with recurrence after primary therapy show that very few die of their disease and that frequently competing causes of death and from prostate cancer-specific death are equally likely upon recurrence of disease [53]. A literature review published in JAMA concluded that “although patients with increasing prostate-specific antigen levels after local treatment without metastatic disease frequently undergo ADT, the benefits of this strategy are not clear… and need to be weighed carefully against substantial risks and adverse effects on quality of life” [54]. This literature review, while indicating the need for additional studies, suggests the usefulness of a risk-stratified approach. This approach includes the incorporation of known risk factors for recurrent aggressive disease to define which patients need more aggressive early therapy and which patients may be able to be spared the adverse events of androgen deprivation at the detection of biochemical recurrence. The elements of risk stratification may include pretreatment PSA, PSA velocity, Gleason score, volume of tumor or stage [55–57], PSA velocity or total value [38, 58], PSA nadir and time to recurrence after therapy [59, 60], and possibly the presence of circulating tumor cells [61], as all of these have been shown to be associated with increased risk of progression or death from prostate cancer. 

## 5. Future Research

Cell-cell signaling and the androgen receptor pathway continue to be appropriate and promising targets for new agents in men with prostate cancer. Currently being investigated are the risks and benefits of the primary use or adjuvant use of agents including somatostatin, or NF-*κ*B ligand (RANKL) receptor antagonists, RNF6-ubiquitiniation inhibitors, aromatase inhibitors, or long-term antiandrogen monotherapy [32, 62–65]. The use of agents to reduce production of dihydrotestosterone in the prevention of prostate cancer is interesting, but so far does not appear to improve the efficacy of complete androgen blockade in men with castration recurrent prostate cancer [66]. The use of 1alpha, 25-dihydroxy vitamin D3 down regulates the expression of prostate-specific membrane antigen in prostate cancer cells [67]. There is a possibility that these agents may improve the efficacy profile of ADT and/or decrease its side effect profile. LHRH antagonists have not been fully studied in all of the scenarios mentioned above but are predicted to have a similar effect to LHRH agonists given with an antiandrogen for the first seven days as their suppression of testosterone is similar [68, 69]. Additional promise lies in pharmaceutical agents such as MDV 3100, an androgen receptor antagonist that inhibits nuclear translocation and DNA binding, demonstrating activity in patients with CRPC [70]. There is high anticipation regarding ongoing research in prostate cancer drugs.

## 6. Summary

The use of ADT has long been recognized as a systemic hormonal treatment for men with prostate cancer, and the specific recommendations for its use continue to evolve. There is increasing evidence over the last five years that continuous long-term ADT causes multiple adverse side effects. Patients need to be evaluated for these risks and should undergo monitoring for hyperlipidemia, coronary artery disease, osteoporosis, and diabetes while castrate. Further insight into the initiation of calcium and vitamin D therapy are now recommended with the initiation of ADT. Intravenous bisphosphonates should now be considered for individuals with metastatic disease or osteoporosis. There is evidence that suggests ADT is warranted in certain clinical situations (see Table 1). However, these recommendations are not firm guidelines and the risks and benefits of ADT must be weighed in any clinical situation. ADT should not be considered primary therapy for men with low-risk prostate cancer. ADT is discouraged in conjunction with prostatectomy unless local lymph nodes are found to be involved. ADT is generally not recommended in patients with low-risk disease with adequate (70 to 79 Gy) conformal (145 Gy), or brachytherapy radiation. In men with a biochemical (PSA-only) recurrence after primary therapy, the timing of the initiation of ADT should be considered carefully. Those patients with high-stage (>2b), high-grade tumors >7, with rapid PSA velocities before and/or after treatment (total PSA > 10 ng/mL before treatment, doubling time <12 months or absolute increase of 2 ng/mL/year before or after treatment), PSA nadir > 0.2 ng/mL, or overall high total PSA (>50 ng/mL) likely benefit from early intervention and possibly from involvement in a clinical trial. Additional situations where ADT is strongly recommended are in a neoadjuvant and setting for 4 to 6 months in men with intermediate risk cancer undergoing radiation therapy or for men with large prostates and low-risk cancer anticipating brachytherapy; in an adjuvant setting for 2 to 3 years in men with high-risk prostate cancer undergoing radiation therapy; in men found to have positive nodes at prostatectomy; in men with symptomatic (obstructing or painful) locally advanced or metastatic disease. ADT may be considered adjuvantly or neoadjuvantly in patients being treated with cryotherapy. Studies regarding use of intermittent ADT, cryotherapy, and androgen receptor pathway modulators are ongoing but appear promising.

## Figures and Tables

**Table 1:** Summary of androgen deprivation indications.

ADT likely unwarranted	ADT still warranted
Localized disease	Advanced disease
(i) Preprostatectomy/postprostatectomy [30]	(i) PSA > 50, PSADT < 12 months [38]
(ii) Prior to EBXRT/Brachytherapy [33–36]	(ii) Prior to EBXRT [43–47]
(iii) No primary treatment [37]	(iii) Local obstructive symptoms and or metastatic disease [50]
(iv) Biochemical recurrence after therapy with PSADT > 12 months [53]	(iv) Lymph node involvement at prostatectomy [49]
	(v) Biochemical recurrence after therapy with high risk of death from prostate cancer [55–57],

Abbreviations. ADT: androgen deprivation therapy; EBXRT: external beam radiation; PSA: prostate-specific antigen; PSADT: prostate-specific antigen doubling time.
